# Host Determinants of Immune Checkpoint Inhibitor Efficacy: Immune, Genetic, Metabolic, and Lifestyle Factors

**DOI:** 10.3390/ijms27104178

**Published:** 2026-05-08

**Authors:** Ali Awada, Nicole Charbel, Sary Faraj, Mohammad Hassan, Duha Awada, Andrea Issa, Wajih Nasr, Sara El Meski, Zuhair Hatahet, Ali Tarhini, Joe Rizkallah, Firas Kreidieh

**Affiliations:** 1 Division of Hematology and Oncology, Department of Internal Medicine, American University of Beirut, Beirut P.O. Box 11-0236, Lebanon; ama221@mail.aub.edu (A.A.); nc47@aub.edu.lb (N.C.); sf67@aub.edu.lb (S.F.); mih34@mail.aub.edu (M.H.); ai80@aub.edu.lb (A.I.); wgn01@mail.aub.edu (W.N.); se153@aub.edu.lb (S.E.M.); zmh21@mail.aub.edu (Z.H.); at110@aub.edu.lb (A.T.); 2 Faculty of Medicine and Medical Sciences, University of Balamand, Tripoli P.O. Box 100, Lebanon; duha.awada@std.balamand.edu.lb; 3 Department of Diagnostic Radiology, American University of Beirut, Beirut P.O. Box 11-0236, Lebanon; jr56@aub.edu.lb

**Keywords:** immune checkpoint inhibitors, host determinants, predictive biomarkers, treatment response, microbiome, metabolism, host genetics, concomitant medications, lifestyle factors

## Abstract

Immune checkpoint inhibitors (ICIs) have revolutionized cancer treatment by enhancing antitumor immune responses; however, clinical outcomes remain highly variable across patients and tumor types. While tumor-intrinsic factors such as PD-L1 expression and tumor mutational burden provide some predictive value, they do not fully explain response heterogeneity. Increasing evidence highlights the critical role of host-related determinants in modulating ICI efficacy. This review provides a comprehensive overview of key host factors, including baseline immune competence, T-cell repertoire diversity, cytokine profiles, and peripheral biomarkers. It further examines the impact of germline genetics, HLA genotype, and epigenetic regulation on immune responses. The role of the gut microbiome and its metabolites is explored, alongside the impact of metabolic status, obesity, nutrition, and lifestyle behaviors. Additionally, the effects of co-medications and comorbidities are discussed. Integrating these host-related factors may improve patient stratification and support the development of personalized immunotherapy strategies.

## 1. Introduction

Immune checkpoint inhibitors (ICIs) are a class of cancer therapies that enhance the body’s own immune system to recognize and eliminate tumor cells [1]. Under normal conditions, immune checkpoints act as regulatory pathways that prevent excessive immune activation and autoimmunity [2]. Tumors can exploit these pathways to evade immune detection by suppressing T cell activity. ICIs have transformed cancer therapy by targeting inhibitory pathways such as programmed cell death protein-1 (PD-1), its ligand PD-L1 and cytotoxic T-lymphocyte-associated protein 4 (CTLA-4), leading to enhanced activation of effector T-cells [2]. By inhibiting these checkpoints, ICIs promote antigen-specific T-cell proliferation, cytotoxic function, and cytokine secretion to drive tumor regression [3]. Clinically, ICIs have demonstrated durable responses in several malignancies, including melanoma, non-small cell lung cancer (NSCLC), renal cell carcinoma and urothelial carcinoma [3]. Approved ICIs include nivolumab and pembrolizumab (anti-PD-1), atezolizumab and durvalumab (anti-PD-L1), and ipilimumab (anti-CTLA-4) [4]. Despite these successes, the overall response rate varies widely across tumor types, ranging from approximately 15% in NSCLC to 40–50% in melanoma [3,5]. This disparity highlights the need to identify predictive markers and mechanisms that influence treatment outcomes.

Clinical responses to immune checkpoint inhibitors are highly variable. Patients with similar tumor histology and expression of established biomarkers may experience different outcomes. Although tumor intrinsic markers such as PD-L1 expression assessed by immunohistochemistry and tumor mutational burden have clinical utility, they account for only part of the variability observed in practice [3,5]. While durable responses are sometimes seen in patients with low or absent expression, treatment failure can occur despite high PD-L1 expression [6]. These findings suggest that determinants extending beyond local tumor characteristics contribute significantly to therapeutic efficacy [5,7].

Host-related factors may influence several steps of the cancer immunity cycle, including antigen presentation, priming and activation of T lymphocytes, expansion of tumor reactive clones, trafficking of immune cells to tumor sites and effector mediated tumor cell killing [7]. A clearer understanding of these influences is necessary to improve patient selection and optimize treatment strategies.

These determinants have a profound effect on ICI outcomes. Germline genetic variation, HLA genotype, and systemic immune status can shape the antitumor immune response [8]. The gut microbiome, through its composition and the production of metabolites such as short-chain fatty acids, tryptophan derivatives, and bile acids, influences systemic immunity and can modulate T-cell activation, dendritic cell maturation, and cytokine production [9]. Disruptions in the microbiome due to antibiotic exposure have been associated with decreased ICI efficacy [8]. Metabolic and lifestyle factors, including obesity, lipid and glucose metabolism, nutritional status, and physical activity, also interact with immune function. Obesity, for instance, has been paradoxically associated with both enhanced and diminished responses in different contexts, reflecting complex immune-metabolic interactions [3,8]. Collectively, these observations highlight the host as an integral mediator of ICI effectiveness.

Although various studies have investigated tumor-specific predictors of immune checkpoint inhibitor response that are specific to the tumor, integrative analyses of host factors are limited. Prior reviews have focused on a single aspect, such as the composition of the microbiome, genetic predisposition, or metabolic influences, frequently in the context of a specific tumor type such as NSCLC or melanoma [5].

In this review paper, we provide a comprehensive analysis of host determinants, including the immune system, genetic and epigenetic factors, the microbiome and metabolome, metabolic and lifestyle influences, and co-medications. Emphasis is placed on translational relevance and factors that can be modified to guide patient selection and therapeutic interventions. By integrating these host-related factors, our review complements tumor-focused analyses and highlights opportunities for optimizing immunotherapy strategies.

## 2. Immune System Factors

### 2.1. Baseline Immune Competence

Baseline immune competence is a critical host determinant of response to immune checkpoint inhibitors (ICIs), as these therapies rely on a functional adaptive immune system to restore antitumor immunity [10]. Peripheral lymphocyte count has emerged as a simple yet informative marker of immune readiness, with baseline lymphopenia consistently associated with reduced ICI efficacy and inferior survival outcomes [11].

T-cell subsets play a particularly central role in mediating ICI responses. Effective tumor cell eradication following immune checkpoint blockade requires adequate numbers of CD8^+^ cytotoxic T cells, while CD4^+^ helper T cells support CD8^+^ T-cell activation, proliferation, and memory formation [12]. Conversely, the expansion of immunosuppressive populations, such as regulatory T cells (Tregs), may dampen antitumor immune responses and limit therapeutic benefit [13]. Alterations in the balance and functionality of these T-cell subsets at baseline can therefore significantly influence ICI responsiveness [14].

### 2.2. T-Cell Repertoire Diversity and Clonal Expansion

T-cell receptor (TCR) repertoire diversity reflects the breadth of antigen recognition and the adaptive immune system’s capacity to respond to tumor-associated neoantigens [15]. Higher baseline TCR diversity has been associated with improved clinical responses to ICIs, suggesting a greater potential for immune recognition following checkpoint blockade [15]. In contrast, restricted repertoire diversity may indicate immune exhaustion, immunosenescence, or chronic antigen exposure, all of which impair therapeutic efficacy [16].

Following ICI treatment, clonal expansion of tumor-reactive T-cell populations is frequently observed and is considered a hallmark of effective immune reinvigoration [17]. However, excessive oligoclonality or dominance of non-tumor-specific clones may reflect dysfunctional immune activation [18]. Thus, both baseline diversity and dynamic clonal expansion patterns are important determinants of ICI outcomes.

### 2.3. NK Cells and Innate Immunity Contributions

Although ICIs primarily target adaptive immune pathways, innate immune cells, particularly natural killer (NK) cells, play an important supportive role in shaping antitumor immunity. NK cells can directly mediate cytotoxicity against tumor cells and contribute to immune priming through cytokine production and crosstalk with dendritic cells [19].

Emerging evidence suggests that functional NK cell activity may enhance ICI responses by promoting antigen release and facilitating T-cell activation within the tumor microenvironment [20]. Conversely, impaired innate immune function or myeloid-driven immunosuppression may limit the effectiveness of checkpoint blockade [21]. Therefore, host innate immune status represents an additional layer of immune competence influencing ICI efficacy.

### 2.4. Cytokine Profile and Systemic Inflammation

The systemic cytokine milieu reflects the balance between immune activation and immunosuppression and has significant implications for ICI efficacy. Elevated levels of pro-inflammatory cytokines, such as interleukin-6 (IL-6) and tumor necrosis factor-α (TNF-α), are commonly associated with chronic inflammation and have been linked to poor responses and increased toxicity during ICI therapy [22].

At the same time, excessive expression of immunosuppressive cytokines, including interleukin-10 (IL-10) and transforming growth factor-β (TGF-β), can inhibit T-cell activation and promote immune evasion [23]. A cytokine environment that favors effective T-cell priming and effector function appears to be a key host determinant of favorable ICI outcomes, underscoring the importance of immune balance rather than inflammation alone [24].

### 2.5. Peripheral Blood Biomarkers

Peripheral blood-based inflammatory and immune biomarkers provide accessible indicators of host immune status and have demonstrated prognostic and predictive relevance in ICI-treated patients. Among these, the neutrophil-to-lymphocyte ratio (NLR) is one of the most extensively studied, with elevated baseline NLR consistently associated with reduced response rates, shorter progression-free survival, and poorer overall survival during ICI therapy [25].

Similarly, increased platelet-to-lymphocyte ratio (PLR) and reduced lymphocyte-to-monocyte ratio (LMR) reflect systemic inflammation and relative lymphocyte suppression, both of which may hinder effective immune checkpoint blockade [26]. Composite indices such as the systemic immune-inflammation index (SII) further integrate multiple immune cell populations and may offer enhanced prognostic value. Collectively, these biomarkers highlight the importance of host immune-inflammation balance in determining ICI efficacy.

## 3. Genetics and Epigenetics

### 3.1. Germline Variants in Immune Checkpoints

Germline genetic variations play a crucial role in shaping an individual’s immune response and can significantly influence the efficacy and toxicity of immune checkpoint inhibitors [27,28]. These inherited genetic differences can affect the expression and function of various immunomodulatory factors within the cancer immunity cycle, thereby impacting both anti-tumor immunity and the propensity for immune-related adverse events (irAEs) [27]. Moreover, the allelic variant (rs16906115) of interleukin 7 (IL-7) has been linked to increased lymphocyte stability after ICI initiation and is considered a major risk factor for ICI-associated irAEs [28,29].

Beyond toxicity, germline variants can also predict ICI efficacy. Studies have shown that pathogenic germline variants (gPVs) in cancer-predisposition genes are associated with improved ICI efficacy and survival across several malignancies, including melanoma [30]. These germline alterations can influence tumor mutational burden (TMB) and the overall molecular phenotype of tumors, thereby impacting their responsiveness to ICIs [31]. For example, patients with Lynch syndrome, who carry pathogenic germline variants in mismatch repair genes (e.g., *MSH2*, *MSH6*, *PMS2*, and *MLH1*), often exhibit higher levels of microsatellite instability, making them more likely to respond to ICIs such as pembrolizumab [31]. The identification of such germline variants plays a crucial role in predicting both the risk of irAEs and the likelihood of favorable responses to ICI therapy [27].

### 3.2. HLA Genotype and Antigen Presentation

The human leukocyte antigen (HLA) system is a crucial component of the adaptive immune system. It plays a fundamental role in presenting tumor antigens to T cells, thereby influencing the recognition and elimination of cancer cells by the immune system. Variations in HLA genotype are increasingly recognized as significant host-related factors that modulate response to ICIs [32]. The diversity of HLA alleles dictates the repertoire of peptides that can be presented, directly impacting the breadth and specificity of the anti-tumor T-cell response.

Specific HLA alleles have been associated with differential outcomes in ICI therapy. For instance, studies have indicated that certain HLA class II genotypes, such as *HLA-DRB4*, may serve as predictive biomarkers for survival following immunotherapy. In patients with metastatic non-small cell lung cancer, the presence of *HLA-DRB4* has been correlated with improved overall survival (OS) [33]. Conversely, specific HLA types have also been implicated in susceptibility to irAEs. The HLA-*DRB4*01 allele, for example, has been linked to an increased likelihood of developing endocrine irAEs in ICI-treated patients [33]. Other studies have explored the association of HLA class I alleles, such as *HLA-A*02*:01*, with ICI efficacy and toxicity, though findings can vary across different cancer types and cohort populations [34]. While some research suggests potential correlations between specific HLA types (e.g., *HLA-B*35, *HLA-DRB111*) and the incidence of particular irAEs like immune-related Pneumonitis, further large-scale, prospective studies are needed to validate these associations and elucidate the underlying mechanisms [34]. The complex interplay between HLA genotype, antigen presentation, and immune response highlights the importance of HLA typing in developing personalized ICI strategies thus optimizing efficacy while minimizing toxicity.

### 3.3. Epigenetic Regulation of Immune Cells

Epigenetic mechanisms involving DNA methylation, histone modifications, and non-coding RNAs, exert significant regulatory control over gene expression without changing the underlying DNA sequence. These regulatory processes are essential for immune cell development, differentiation, and function, and their dysregulation can significantly impair the host’s anti-tumor immune response, consequently impacting ICI efficacy [35,36]. Epigenetic modifications can influence the tumor microenvironment (TME) by altering the expression of immune checkpoint molecules, antigen presentation machinery, and various cytokines and chemokines that govern immune cell chemotaxis, infiltration and activity [37,38].

Evidence based research highlights the importance of targeting epigenetic mechanisms to enhance ICI responsiveness. Epigenetic modulators, for instance, can prime the immune system by augmenting tumor immunogenicity and reversing immunosuppression within the TME [39,40]. DNA methyltransferase inhibitors (DNMTis) and histone deacetylase inhibitors (HDACis) are two classes of epigenetic drugs that have demonstrated considerable therapeutic potential in both preclinical models and clinical trials. These agents can upregulate the expression of tumor antigens, major histocompatibility complex (MHC) molecules, and co-stimulatory ligands on cancer cells, rendering them more recognizable to T cells [41]. Furthermore, epigenetic drugs can directly impact immune cells by promoting the differentiation of anti-tumor immune cells (e.g., cytotoxic T lymphocytes) and inhibiting immunosuppressive populations (e.g., myeloid-derived suppressor cells, regulatory T cells) [42]. The reversible nature of epigenetic modifications makes them potential targets for therapeutic interventions aimed at improving outcomes with ICIs. Consequently, combination strategies incorporating epigenetic drugs with ICIs are being actively investigated to overcome resistance mechanisms and enhance clinical responses across multiple cancer types [43].

### 3.4. Pharmacogenomics and Personalized Immunotherapy

Pharmacogenomics, the study of how an individual’s genetic makeup influences their response to pharmacological agents, is becoming increasingly critical in the era of personalized immunotherapy. By identifying germline genetic variations that impact drug metabolism, pharmacodynamics, and toxicity, pharmacogenomics offers a robust approach to optimizing ICI therapy [29].

The observed heterogeneity in patient responses to ICIs highlights the necessity for predictive biomarkers that can guide treatment selection and mitigate adverse events. Germline genetic host factors serve as crucial predictive biomarkers in immuno-oncology. Inherited genetic variations, for example, can influence the risk of developing irAEs, with some studies suggesting that autoimmune genetic risk may be a significant indicator of both ICI efficacy and toxicity [28]. Pharmacogenomic studies aim to identify single nucleotide polymorphisms (SNPs) or other genetic variants that correlate with improved response rates, prolonged survival, or increased susceptibility to specific irAEs. This deeper understanding enables more precise patient stratification, allowing clinicians to identify individuals most likely to benefit from ICI therapy while minimizing severe toxicities in others [44]. Ongoing research is actively exploring the utility of polygenic risk scores, which integrate multiple genetic variants, to predict ICI discontinuation due to irAEs [45]. Ultimately, the effective integration of pharmacogenomics into clinical practice holds promising results towards achieving more personalized immunotherapy.

Importantly, the influence of host-related determinants on ICI efficacy varies across cancer types, reinforcing the need for tumor-specific predictive frameworks [46,47,48]. Highly immunogenic tumors such as melanoma and non-small cell lung cancer (NSCLC) demonstrate stronger associations between host immune competence, T-cell repertoire diversity, and microbiome composition and treatment response [47,49,50]. In contrast, immunologically “cold” tumors such as pancreatic cancer and certain colorectal cancers often show attenuated host immune effects and may require combination strategies to achieve clinical benefit [51]. Collectively, these observations highlight the importance of integrating both host genomic architecture and tumor immunobiology to advance truly personalized and cancer-type–adapted immunotherapy strategies.

## 4. Microbiome and Metabolome

### 4.1. Gut Microbiota Composition

**Figure 1:**
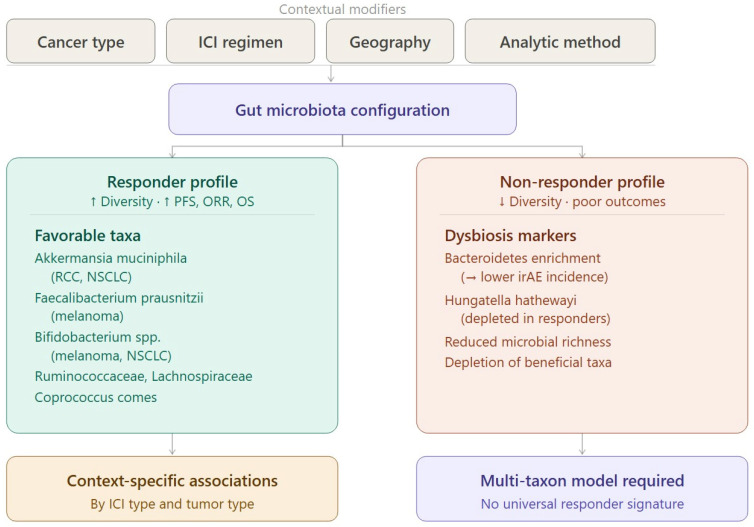
Context-Dependent Gut Microbiota Profiles in ICI Responders and Non-Responders.

ICI responders typically exhibit a more favorable baseline gut microbiota configuration [52]. Those configurations are generally characterized by higher microbiome diversity, or by a gut richer in certain taxa with immunostimulatory or barrier-supportive functions. In comparison, non-responders frequently present a gut with reduced diversity and depletion of beneficial taxa [52]. These associations are summarized in Figure 1, highlighting the contrasting microbiota profiles of responders and non-responders, as well as the influence of context-specific factors.

However, no single microbiome composition has been identified as a “universal responder”, as composition–response associations depend on the interaction between multiple parameters including cancer type, ICI regimen, geography, and analytic method [53,54,55].

Greater baseline microbial diversity was associated with better ICI outcomes in some cohorts, most notably progression free survival [54,56]. However, other reviews have not found a significant association between high microbiome diversity and ICI efficacy across cancer types, suggesting that the microbial community structure may be more important than diversity alone [54,56].

Many bacterial taxa have been linked to a better ICI response. Importantly, these associations appear to be context-specific, varying by ICI type and tumor type, indicating that microbial predictors appear partly context-specific to cancer type and checkpoint regimen [53]. Examples of species mentioned were *Akkermansia muciniphila*, which was associated with improved PFS in RCC, and higher ORR, OS and improved PFS in NSCLC [56]. *Faecalibacterium prausnitzii* is another major recurring responder-associated taxon, particularly in melanoma patients [53,54]. *Bifidobacterium* species were associated with improved outcomes in melanoma and NSCLC [54,56]. Other taxa that were linked to better ICI response are Ruminococcaceae, Lachnospiraceae and *Coprococcus comes* [53,54,56], while depletion of *Hungatella hathewayi* was noted in treatment responders [53]. On the other hand, poor ICI efficacy was associated with certain microbiome configurations such as Bacteroidetes, which were linked to poorer ICI efficacy but to a lower irAE incidence [56].

Overall, current evidence supports gut microbiome configuration as a relevant biomarker of ICI responsiveness and a potentially modifiable host factor, although a multi-taxon model and context specific analysis are required to complete the understanding of all factors interplaying in the balance influencing the degree of ICI efficacy [53,54,56].

### 4.2. Microbial Metabolites

Beyond the fact that microbiome composition influences the response to ICI treatment, microbial metabolites represent key factors influencing the host immunity by modulating immune cell differentiation, metabolic pathways, and inflammatory signaling within the tumor microenvironment, thereby influencing ICI responses [54,57]. Three groups of metabolites are of interest in this regard.

The first group is the short chain fatty acids (SCFAs), produced via fermentation of dietary fibers, and include acetate, propionate, and butyrate among others. The influence of SCFAs on the immune response is well studied, and their effect has been documented in epigenetic regulation, receptor-mediated signaling, particularly through certain G-protein coupled receptors, and T-cell differentiation [54]. SCFA production is strongly associated with beneficial microbial taxa frequently enriched in ICI responders, including *Faecalibacterium*, Ruminococcaceae, and Lachnospiraceae [54,58]. These bacteria are major butyrate producers and are thought to contribute to enhanced immune activation and improved responses to ICI therapy, particularly checkpoint blockade [54].

The second group is the tryptophan-derived metabolites. These by-products activate the aryl hydrocarbon receptor (AhR), a transcription factor that regulates immune cell differentiation and mucosal immune homeostasis and which plays a role in regulatory T-cell differentiation, Th17 responses, cytokine production, and epithelial barrier integrity [54]. Dysregulation of tryptophan metabolism has been linked to several cancer-related immune processes, including tumor immune evasion and systemic inflammation [54,59].

The third group is the bile metabolites, specifically the secondary bile acids, which have been shown to interact with many host receptors, including FXR (farnesoid X receptor), TGR5 (GPBAR1), VDR and RORγt receptors. The signaling pathways influenced by these interactions are involved in multiple immune processes, including dendritic cell function, T-cell differentiation, inflammatory cytokine production, CD8^+^ T-cell activation and antigen presentation [54].

As discussed, microbial activity, through its diverse metabolite production, acts as a functional mediator linking microbiota composition to systemic immune regulation, leading to regulation of both the tumor and gut microenvironment and a possible influence over ICI response [54,58,59,60].

### 4.3. Microbiome Modifying Agents and Mechanistic Effects on ICI Response

The gut microbiota is significantly influenced by concomitant medication use, which can alter ICI responses (see section for clinical implications) [54,56].

Among microbiome-modifying exposures, antibiotics are key disruptors of gut microbial composition [56]. Their impact is primarily mediated through depletion of beneficial taxa and reduced microbial diversity, leading to impaired immune modulation [56]. Additionally, prolonged avoidance of antibiotics from the timing of ICI initiation was linked to longer overall survival, indicating a clinically relevant temporal factor of the disruption of the gut flora.

Proton pumps inhibitors (PPIs) are another important medication class with notable effects over the gut flora through gastric acid suppression. PPI-induced changes in microbial composition include increased oral taxa and reduced diversity, which may impair antitumor immunity [56].

Among microbiome-directed interventions, fecal microbial transplantation is the strongest proof-of-concept strategy for overcoming an unfavorable microbiome state. FMT from immunotherapy-responsive donors to recipients was shown to be able to overcome resistance to anti-PD-1 therapy in some melanoma patients not responding to initial ICI treatment [54,56].

Research on probiotics and microbiome-targeted biotic interventions related to ICI response has been more limited compared to antibiotics and FMT, and routine use of probiotics is still not yet established as an adjunct to ICI therapy [54,56]. These classes of medications represent a more focused approach toward reconfiguring the microbial environment and consequently enhancing the response to ICIs. For example, probiotics such as *Akkermansia muciniphila* and *Faecalibacterium prausnitzii* showed enhancement of ICI efficacy in preclinical trials [54]. *Clostridium butyricum* CBM588 significantly improved PFS and ORR when added to ICI treatment, possibly through enhancement of metabolite production and enrichment of the microbiome with beneficial taxa like Ruminococcaceae [56].

### 4.4. Dietary and Lifestyle Influences

In addition to the effect of medications on the gut flora, dietary habits and lifestyle factors could have a significant effect on the composition of the human microbiome, influencing ICI efficacy [56]. In particular, diet and nutritional patterns affect the diversity and metabolic activity of gut microbiota as higher dietary fiber intake was linked to better ICI outcomes [56]. Preclinical studies have observed impaired responses to ICI treatment associated with low fiber diets, in contrast to a possibly enhanced immune activity in fiber-rich diets [56]. Beyond diet alone, lifestyle variables such as physical activity, smoking, alcohol consumption, and sleep may play a role in the regulation of gut microbiota composition, leading to changes in the local and systemic immune responses [55].

### 4.5. Mechanistic Links to T-Cell Activation

Beyond microbiome composition and metabolites in enhancing the efficacy of ICI therapy, the cellular mechanisms linking immunotherapy and microbiome have been reviewed extensively [52,54,56,61]. The key players in this interaction are dendritic cells, particularly their role in the process of antigen presentation. Specifically, the activation and maturation of dendritic cells have been observed to be influenced by certain commensals, engaging TLRs and other innate sensors on intestinal and tumor-associated DCs, leading to increased production of cytokines such as IL-12, IFN-γ and other pro-inflammatory cytokines, and increased expression of key receptors in the antigen presentation process such as CD80/86 and MHC. This enhances cross-presentation of tumor-related antigens [54,56,61]. DC maturation is essential for effective CD4^+^ and CD8^+^ priming [54,56]. In several mouse tumor models and human correlative data, specific microbiota configurations were linked to DC maturation states and intratumoral gene signatures that support Th1-like and CD8^+^ T cell-mediated antitumor immunity under PD-1 blockade [56]. Overall, the evidence shows an early influence of microbiome composition in the preparation of the microenvironment with a primed T-cell pool by a mature DC population [54,56,61].

In addition to effects on immune priming, a direct effect of the microbiota and its metabolites has been demonstrated in several models on CD8^+^ T cells (3,4,7). Enhanced proliferation, IFN-γ production, and cytotoxicity of CD8^+^ T cells were observed in tumor-bearing mice that were exposed to certain microbial gut taxa, indicating an active effect of the gut microbiota on the immune response rather than a passive one [53,54,59].

Examples of studied mechanisms of these beneficial effects include the inosine-mediated augmentation of IFN-γ production in CD8^+^ T cells through adenosine A2A receptor signaling in the presence of appropriate co-stimulation [56,59]. Additionally, the enhancement by bacterial tryptophan metabolites of CD8^+^ T cell function and survival in tumor models via AhR-dependent transcriptional modulation demonstrates the metabolic and epigenetic role of the microbiome [54,56,59]. In addition, SCFAs were shown to modulate cytokine output and co-stimulation in DCs, indirectly biasing T cell priming, while also influencing both effector and regulatory T-cells [54,56]. Secondary bile acids act as signaling molecules regulating Th17 cells, regulatory T cells, and cytotoxic responses through interaction with downstream receptors involved in multiple immune processes [54,56].

The role of the gut microbiome in controlling T-cell trafficking and infiltration within the microenvironment is also critical, as the efficacy of ICI therapy is largely determined by the infiltration of immune cells in the tumor bed [52,54]. Certain commensal communities were observed to upregulate the production of cytokines such as CXCL9 and CXCL10 involved in creating gradients that influence CD4^+^ and CD8^+^ T-cell recruitment in the gut and tumor tissues [54,61]. There is also observational evidence that certain taxa configurations increase the infiltration of T-cells in the tumor environment, augmenting the response to immunotherapy by enhancing the efficacy of anti-PD-L1 therapy [52,61]. These mechanisms contribute to a pro-inflammatory, T-cell inflamed tumor microenvironment, which is strongly associated with improved ICI response [52,54,61].

## 5. Metabolic and Lifestyle Factors

### 5.1. Obesity, Adipokines, and the Obesity Paradox

Obesity results in significant metabolic and inflammatory alterations that influence both tumor biology and host immune responses. Adipose tissue acts as a central endocrine organ capable of modulating immune cell activity and systemic inflammation through the secretion of adipokines, cytokines, and other bioactive mediators [62]. Chronic obesity is associated with elevated circulating levels of multiple proinflammatory cytokines including tumor necrosis factor α and interleukin 6, both of which contribute to immune dysregulation and tumor progression [63]. This concept has gained attention due to the “obesity paradox”, where obese patients exhibit improved outcomes following immune checkpoint blockade. Accordingly, a higher body mass index (BMI) has been associated with a longer overall survival and a progression free (PFS) survival in patients receiving ICIs [64]. Proposed mechanisms include that obesity induces chronic immune activation and leads to T-cell exhaustion characterized by increased expression of inhibitory receptors such as PD-1. This immune phenotype may cause tumors to become more susceptible to PD-1/PD-L1 blockade, thereby increasing the responsiveness to ICIs [63]. However, interpreting this paradox requires caution. The measurement of BMI alone is an imperfect reflection of metabolic health, as it fails to discriminate between lean mass, visceral adiposity, and sarcopenia. Emerging evidence suggests that the body composition and metabolic status of the patient may serve as better informative predictors of immunotherapy outcomes than BMI alone, since factors like sarcopenic obesity and visceral fat accumulation can impact systemic inflammation and immune competence [65]. Integration of metabolic biomarkers and body composition assessments offers a more precise understanding of the relationship between obesity and immunotherapy response [63].

### 5.2. Glucose and Lipid Metabolism in Immune Regulation

Metabolic pathways that regulate glucose and lipid utilization play a central role in immune regulation and cancer progression. Tumor cells undergo several metabolic alterations that increase their glucose uptake and their glycolytic activity, creating a metabolically competitive microenvironment [66]. This type of competition limits the nutrient availability for immune effector cells, specifically cytotoxic T lymphocytes, thus impairing their activation and ultimately their function [67]. Moreover, systemic metabolic disorders including hyperglycemia and diabetes can further lead to the disruption of immune homeostasis. Chronic hyperglycemia is associated with an overall increased systemic inflammation, immune dysfunction, and enhanced expression of inhibitory immune checkpoint molecules on T cells [68]. Consequently, these alterations weaken antitumor immunity and may reduce ICI effectiveness [67]. Additionally, lipid metabolism was also found to contribute to overall tumor progression and immune regulation. Tumor cells also alter their lipid metabolic pathways in order to support the required rapid proliferation, immune evasion, and metastatic dissemination. Dysregulated lipid metabolism may modulate immune cell differentiation and promote immunosuppressive phenotypes within the TME, such as tumor-associated macrophages and regulatory T cells [69,70]. These findings highlight the therapeutic potential of targeting metabolic pathways involved in lipid synthesis and oxidation to enhance the effectiveness of cancer immunotherapy [71].

### 5.3. Exercise, Circadian Rhythm, and Immune Function

Lifestyle factors, including circadian regulation and physical activity, influence host immune competence. The circadian system is usually responsible for the coordination of the daily oscillations in immune cell trafficking, cytokine production, and inflammatory signaling [72]. Disruption of circadian rhythms, including irregular sleep patterns, light exposure at night, or shift work, can impair immune surveillance and alter the tumor immune interactions [73]. Moreover, circadian regulation is closely related to lifestyle behaviors in particular diet and physical activity, which alters systemic metabolism and immune responses. Experimental studies suggest that circadian misalignment may alter the TEM and potentially diminish the effectiveness of antitumor immune responses [73]. Furthermore, physical activity may enhance antitumor immunity by promoting and increasing the infiltration of cytotoxic T cells along with natural killer cells into the tumor [73]. While lifestyle modifications improving circadian regulation and promoting physical activity may complement immunotherapy strategies, direct clinical evidence in patients receiving ICIs remains limited [73].

### 5.4. Nutrition and Micronutrients

Nutritional status of the patient represents another significant determinant that may be modified, hence influencing immunotherapy outcomes. It affects immune competence, the extent of systemic inflammation, and the metabolic regulation, all of which are required for effective antitumor immune responses [74,75]. Clinical evidence suggests that inadequate nutritional status is linked to inferior outcomes in cancer patients treated with ICIs, thus highlighting the importance of metabolic health in determining treatment response [76]. Moreover, diet composition may also alter the immunotherapy efficacy via the modulation of various metabolic pathways and through the gut microbiome. Diets rich in fiber, fruits, vegetables, and other plant based nutrients were shown to be associated with the promotion of microbial diversity and the production of metabolites that support immune activation, potentially enhancing responses to immunotherapy [77]. Additionally, certain dietary bioactive compounds, such as polyphenols, vitamins, and fatty acids, demonstrated the effective capacity to influence tumor metabolism, angiogenesis, and immune signaling in certain experimental cancer models [78]. However, clinical evidence supporting the use of such specific nutritional supplements during the course of the immunotherapy remains limited, emphasizing the need for future clinical studies to determine their impact on ICI outcomes.

## 6. Co-Medications and Comorbidities

### 6.1. Medications Affecting ICI Efficacy

Growing evidence indicates that standard medications can significantly affect the efficacy and toxicity of immune checkpoint inhibitors. Antibiotics are the drug class most often linked to these effects, with several studies showing that antibiotic use, especially when given before or shortly after starting ICI therapy, is associated with reduced efficacy [79,80,81]. In a systematic review and meta-analysis concerning non-small cell lung cancer (NSCLC), antibiotic exposure prior to or during ICI therapy was correlated with poorer survival outcomes, with the most pronounced effect observed during the early exposure period [82]. Similarly, in a prospective multicenter cohort study, prior antibiotic use was associated with significantly reduced overall survival and increased rates of primary refractory disease. Conversely, concurrent antibiotic use was not associated with these outcomes, underscoring the importance of timing [83]. Large real-world datasets reveal similar associations; for example, a population-based cohort of older adults in Ontario initiating ICIs, where pre-ICI antibiotic use was linked to reduced overall survival (OS) following multivariable adjustments [84].

These effects are mainly due to antibiotic-induced disruption of the gut microbiome, which is crucial in shaping antitumor immune responses [85,86]. These effects are thought to arise from antibiotic induced disruption of the gut microbiome and its immune-active metabolites, which are key regulators of systemic and intratumoral antitumor immunity. For instance, a prospective NSCLC stool profiling study demonstrated that the administration of antibiotics prior to ICI was associated with reduced baseline alpha diversity and depletion of taxa linked to a favorable overall response rate, progression-free survival, and overall survival [87]. Preclinical mechanistic studies indicate that, beyond mere association, ICI can facilitate the movement of specific commensals to lymphoid organs and tumors. This translocation enhances dendritic-cell activation and promotes effector CD8+ T-cell responses. Additionally, antibiotics use may interfere with this process, leading to reduced antitumor immunity [88].

Proton pump inhibitors (PPIs) have also been linked to decreased ICI effectiveness in several retrospective studies. Their effects may also involve microbiome-related mechanisms (Section 4.3), in addition to potential impacts on immune activation [81,89,90]. Furthermore, PPIs are recognized contributors to clinically significant drug–drug interactions, which may further undermine the efficacy of anticancer treatments [89].

Systemic corticosteroids are of particular concern due to their strong immunosuppressive effects. Baseline steroid use has consistently been linked to worse outcomes in patients treated with PD-1 or PD-L1 inhibitors, especially in patients with non-small-cell lung cancer [81,91]. However, emerging prospective data suggest that the timing and indication for steroid use are critical factors; steroids used to manage immune-related adverse events (irAEs) may not consistently negate the benefits of ICIs [92,93].

Data on nonsteroidal anti-inflammatory drugs (NSAIDs) remain limited and heterogeneous. While preclinical evidence indicates that cyclooxygenase inhibition may boost antitumor immunity, clinical studies have shown mixed results, with some suggesting no significant effects and others raising concerns about their impact on toxicity profiles [85,86].

### 6.2. Chronic Comorbidities

Chronic comorbidities are increasingly acknowledged as important factors affecting ICI outcomes, especially in real-world populations. Diabetes mellitus has been linked to chronic inflammation, immune dysregulation, and altered T-cell metabolism, all of which may impact antitumor immune responses [94]. Observational studies indicate that diabetic patients might have different survival outcomes and irAE patterns, although findings are still inconsistent and could be influenced by antidiabetic medication use [94].

Cardiovascular disease (CVD) is another significant comorbidity that may impact ICI therapy. Patients with CVD often take multiple cardiovascular medications and may have underlying immune senescence or endothelial dysfunction, which could influence treatment effectiveness and toxicity [86]. ICIs have also been associated with rare but serious cardiovascular irAEs, which further complicates treatment in this group [95].

Studies focusing on older adults with cancer highlight the high prevalence of comorbidities and polypharmacy, which are linked to increased drug–drug interactions, treatment disruptions, and adverse outcomes [96]. These findings highlight the limited external validity of clinical trials, which often exclude patients with significant comorbidities burdens.

### 6.3. Interactions Between Drugs, Comorbidities, and Immune Response

Concomitant medications and chronic comorbidities probably interact in complex ways to influence immune responses and clinical results during ICI therapy. Antibiotic-related microbiome disruption might have a stronger impact on patients with diabetes, advanced age, or frailty, increasing immune dysfunction [86]. Similarly, baseline steroid use may reflect both the effects of immunosuppressive drugs and the severity of the underlying disease, complicating causal inference [85].

Large pan-cancer analyses show that concomitant medications affect not only survival but also the incidence and severity of irAEs, which have been linked to improved survival in multiple studies [92,94,95]. These findings suggest that immune activation sufficient to induce toxicity may also reflect effective antitumor immunity.

Recent systematic reviews and large cohort studies highlight that the timing, duration, and reasons for medication exposure are key factors influencing their effect on ICIs [86,90]. Acute peri-treatment exposures may have different biological effects compared to chronic baseline use, especially during early immune priming. Overall, these findings emphasize the importance of integrated approaches that consider medications, comorbidities, and immune dynamics when assessing ICI outcomes. Key host-related determinants influencing response to immune checkpoint inhibitors are summarized in Table 1.

## 7. Translational Applications: Host Targeted Interventions

### 7.1. Microbiome-Based Therapeutic Strategies

#### 7.1.1. Fecal Microbiota Transplantation (FMT) and Immunotherapy Synergy

The gut microbiome has emerged as a key host factor influencing immunotherapy responses [97]. Multiple studies have demonstrated that microbiome composition and diversity are strongly associated with ICI response, with dysbiosis linked to poorer outcomes and specific taxa associated with improved therapeutic efficacy (see Section 4). Mechanistically, these effects are mediated through modulation of immune signaling, microbial metabolite production, and interactions with host immune pathways (Section 4).

Microbiota transplantation can enhance immune activation and increase the abundance of immune cells such as CD4+ and CD8+ T lymphocytes [97].Clinical trials combining Fecal Microbiota Transplantation (FMT) with anti-PD-1 or anti-CTLA-4 therapies have reported encouraging results, including increased objective response rates and prolonged progression-free survival in patients with melanoma, renal cell carcinoma, and gastrointestinal cancers, compared to ICI therapy alone [97].

Despite these promising findings, several challenges remain for clinical implementation. The success of FMT depends on factors such as donor microbiota composition, recipient microbiome characteristics, and treatment timing relative to immunotherapy.

#### 7.1.2. Clinical Trials of FMT in Cancer Immunotherapy

Several clinical trials have investigated the use of fecal microbiota transplantation (FMT) from responders to anti-PD-1 therapy as a strategy to enhance treatment efficacy in non-responders. Early landmark studies demonstrated proof-of-concept in immunotherapy-refractory melanoma. For example, a phase I trial showed that FMT combined with anti-PD-1 therapy induced objective responses in a subset of patients, with 3 out of 10 achieving clinical responses, including one complete response, alongside significant modulation of the gut microbiome and enhanced immune activation within the tumor microenvironment [98]. Similarly, another phase II study (NCT03341143) reported that approximately 40% of patients achieved disease control following FMT from PD-1 responders, with an objective response rate of around 20%, associated with successful microbiota engraftment and increased CD8^+^ T-cell activity [99].

Building on these findings, the trial identified as NCT03772899 focused on FMT from healthy donors combined with PD-1 inhibitors (nivolumab/pembrolizumab) in untreated advanced melanoma patients, reporting a 65% objective response rate, including 20% complete responses, with FMT being safe and leading to beneficial microbiome changes [100]. Another trial, the phase II FMT-LUMINate trial (NCT04951583), examined FMT plus anti-PD-1 therapy in patients with non-small cell lung cancer (NSCLC) and melanoma, achieving high response rates of 80% in NSCLC and 75% in melanoma, while also altering gut microbiota by reducing deleterious taxa linked to better outcomes [101]. The trial NCT04264975 assessed FMT from anti-PD-1 responders in patients with advanced solid cancers who were refractory to anti-PD-1 therapy, resulting in a 46.2% disease control rate and inducing microbiota changes that increased cytotoxic T-cell activity [102]. Additionally, the ongoing trial NCT05251389 is evaluating the clinical benefit of FMT from ICI responders versus non-responders in anti-PD-1-refractory melanoma patients [103]. Another trial, NCT04130763, focused on FMT from healthy donors combined with anti-PD-1 therapy in advanced gastrointestinal cancers resistant to PD-(L)1 therapy, reporting a 20% objective response rate and correlating donor-derived microbes with immune activation [104]. Lastly, the trial MR3622010054 investigated FMT combined with PD-1 inhibitor rechallenge and chemotherapy in advanced NSCLC with secondary PD-1 resistance, achieving a 29.6% objective response rate and demonstrating increased microbial diversity and CD8^+^ T-cell infiltration in responders [105]. Key clinical trials evaluating the therapeutic potential of FMT in combination with immune checkpoint inhibitors are summarized in Table 2.

Collectively, these studies highlight consistent patterns in responders, including enrichment of beneficial taxa such as *Akkermansia muciniphila*, increased microbial diversity, and enhanced antitumor immune responses characterized by increased cytotoxic T-cell activity and reduced immunosuppressive mechanisms, supporting the gut microbiome as a key modulator of immunotherapy efficacy.

#### 7.1.3. Microbiome Modulation in Treatment-Related Toxicity

Beyond its role in enhancing immunotherapy efficacy, clinical trials have also investigated FMT for the management of treatment-related toxicity and complications across various medical conditions, particularly in oncology, immune-mediated colitis (IMC), and graft-versus-host disease (GvHD). In oncology, FMT has demonstrated potential benefits in mitigating chemotherapy- and immunotherapy-induced toxicities, restoring gut microbiota diversity, and reducing immune-related adverse events (irAEs) such as colitis, with clinical improvement observed in treatment-induced colitis and a reduction in colonic CD8+ T-cell infiltration [106,107,108].

### 7.2. Antibiotics and Microbiota Disruption

Antibiotic use has profound effects on the gut microbiota and immune system, with significant implications for human health. Antibiotics disrupt the composition and diversity of the gut microbiota, leading to dysbiosis, which can impair immune homeostasis and increase susceptibility to infections and immune-mediated diseases [109,110]. This disruption is associated with reduced production of critical microbial metabolites, such as short-chain fatty acids (SCFAs), which are essential for maintaining immune balance [111,112]. Additionally, antibiotic-induced dysbiosis can promote the proliferation of antibiotic-resistant strains, further complicating health outcomes [113,114].

The immune system is intricately linked to gut microbiota, with the latter playing a crucial role in the development and modulation of both innate and adaptive immunity. Antibiotic exposure can alter immune responses, such as reducing vaccine efficacy and impairing the protective effects of the microbiota against pathogens [113,115]. In some cases, antibiotics have been shown to increase pro-inflammatory responses and shift immune cell phenotypes, as observed in studies on vaccine responses and systemic immunity [115]. Early-life antibiotic exposure is particularly concerning, as it can disrupt the immature microbiota, leading to long-term immune dysregulation and increased risk of diseases like inflammatory bowel disease and colitis [116,117].

Strategies to mitigate these adverse effects include the use of probiotics, prebiotics, and microbiota-targeted therapies to restore microbial balance and enhance immune function [118]. Additionally, reducing unnecessary antibiotic use and exploring alternative treatments, such as vaccines, can help minimize the development of antimicrobial resistance and its impact on the microbiome [114]. Overall, understanding the complex interplay between antibiotics, microbiota, and immunity is critical for developing effective interventions to preserve health and combat antibiotic resistance.

### 7.3. Metabolism

Metabolic reprogramming plays a critical role in regulating tumor and immune cell function, including immune checkpoints pathways [119]. Specifically, tumor cells deplete nutrients (glucose, tryptophan, arginine) and generate toxic metabolites (lactate, adenosine, kynurenine) that impair T cell activation and promote exhaustion, limiting the therapeutic effect of ICIs [120].

### 7.4. Cytokine Modulation

Within the tumor immune microenvironment, multiple immune cell populations influence treatment outcomes. Cells such as neutrophils, myeloid-derived suppressor cells (MDSCs), natural killer (NK) cells, dendritic cells, mast cells, and regulatory T cells (Tregs) can either promote antitumor immunity or contribute to immunosuppression depending on their functional state and the signals present in the tumor microenvironment [121].

Cytokine signaling pathways influence immune cell recruitment, activation, and differentiation, thereby affecting antitumor immunity [122]. Several cytokines, including interferons and interleukins, have been investigated as therapeutic agents capable of enhancing immune activation and promoting antitumor immune responses [122].

Mechanistically, a “push–pull” framework applies: locally amplifying Th1/CTL-supporting signals (IL-2, IL-12, IL-15, type I/II interferons) while attenuating suppressive circuits (TGF-β, IL-6/STAT3, IL-8, IL-10) [24]. While early-phase trials with IL-2 formulations and IL-15 super agonists show “promising efficacy data” in combination with ICI [123], broader clinical outcomes remain unclear. De Luca et al. notes that “results in human clinical trials are less clear” despite strong preclinical support, attributing this to systemic toxicity, network redundancy, and counter-regulatory feedback [124]. Thus, evidence supports cytokine modulation’s mechanistic importance, but clinical efficacy requires further optimization.

Microbiota-based therapies may also influence cytokine signaling pathways by stimulating immune cells involved in antitumor responses. Certain bacterial species have been shown to activate dendritic cells and subsequently stimulate tumor-specific CD8+ cytotoxic T cells, thereby enhancing antitumor immunity [97].

### 7.5. Biomarkers for Patient Stratification

One of the major challenges in cancer immunotherapy is the variability in patient responses to immune checkpoint inhibitors. Identifying predictive biomarkers is therefore essential for selecting patients who are most likely to benefit from immunotherapy [122]. Several biomarkers have been investigated as predictors of response to immune checkpoint blockade. PD-L1 expression on tumor cells is among the most widely used biomarkers in clinical practice. Higher levels of PD-L1 expression have been associated with improved responses to PD-1 and PD-L1 inhibitors [122].

Two other FDA-approved biomarkers are also routinely used for ICI patient stratification: tumor mutational burden (TMB), and microsatellite instability (MSI), which are commonly used to guide patient selection for immune checkpoint blockade [125]. However, these biomarkers have limitations, as they only partially capture the complexity of treatment response [125]. Emerging biomarkers under investigation include gene expression signatures, tumor-infiltrating lymphocytes, circulating tumor DNA, gut microbiome composition, and immune cell subsets, which may further improve predictive accuracy for immunotherapy response [126].

### 7.6. Future Research Directions

Despite significant progress in cancer immunotherapy, many patients either fail to respond to immune checkpoint inhibitors or develop resistance during treatment. Future research should therefore focus on identifying novel therapeutic strategies and improving the understanding of the mechanisms underlying immunotherapy resistance [122].

Bai et al. emphasized that multifactorial predictive markers integrating tumor–host interactions represent a key direction for precision immuno-oncology [125]. Recent advances also highlight the role of artificial intelligence-driven multi-omics analysis for dynamic patient stratification and biomarker discovery [126].

Additionally, combination immunotherapy strategies and epigenetic approaches are increasingly investigated to address complex mechanisms of resistance to immune checkpoint inhibitors [127].

Emerging immune checkpoint targets include lymphocyte activation gene-3 (LAG-3), T cell immunoreceptor with immunoglobulin and tyrosine-based inhibitory motif domains (TIGIT), and T-cell immunoglobulin and mucin-domain containing-3 (TIM-3), which represent promising therapeutic targets currently under investigation [126].

Microbiome-based approaches, including fecal microbiota transplantation, also represent emerging strategies to enhance the efficacy of immune checkpoint inhibitors and potentially mitigate treatment-related adverse effects [97].

Emerging technologies are expected to advance the understanding of host-related determinants of immune checkpoint inhibitor efficacy. Single-cell omics enables high-resolution profiling of immune cell populations, while PROTAC-based strategies allow targeted degradation of key regulatory proteins. In parallel, advanced proteomic approaches provide insights into signaling networks governing immune responses. Together, these tools may facilitate the identification of novel therapeutic targets and improve personalized immunotherapy strategies [128,129,130].

## 8. Conclusions

Host-related factors are central determinants of ICI efficacy. Key determinants include baseline immune competence, genetic and epigenetic variation, gut microbiome composition, metabolic status, and the impact of co-medications and comorbidities. Importantly, several of these factors are potentially modifiable. Interventions targeting the microbiome, optimizing metabolic health, improving nutrition, and minimizing unnecessary use of antibiotics or corticosteroids may enhance treatment responses. Clinically, integrating host-related biomarkers with tumor-specific factors can improve patient stratification and guide personalized therapy. Translational approaches that combine ICIs with microbiome modulation, metabolic interventions, or immune-targeted strategies hold promise for overcoming resistance and maximizing therapeutic benefit.

## Figures and Tables

**Table 1:** (A) Immune and Genetic Determinants Influencing Response to Immune Checkpoint Inhibitors. (B) Microbiome, Metabolic, Lifestyle, and Pharmacologic Determinants Influencing Response to ICIs.

Category	Factor	Effect on ICI Response	Mechanism	Clinical Relevance
(A)				
Immune System	High CD8^+^ T-cell levels	↑ Improved response [10,12,14]	Enhanced cytotoxic tumor cell killing	Predictive biomarker
Immune System	High neutrophil-to-lymphocyte ratio (NLR)	↓ Reduced response [25]	Systemic inflammation and immune suppression	Prognostic marker
Immune System	Expansion of regulatory T cells (Tregs)	↓ Reduced response [13]	Suppression of effector T-cell activity	Potential therapeutic target
Immune System	Favorable cytokine profile (low IL-6, balanced immune signaling)	↑ Improved response [22,24]	Promotes effective T-cell activation and function	Potential biomarker
Genetics	HLA diversity	↑ Improved response [32,34]	Broader antigen presentation to T cells	Patient stratification
Genetics	Germline variants (e.g., IL-7 SNPs, MMR genes)	↑/↓ Variable [27,31]	Modulation of immune response and tumor immunogenicity	Predictive of efficacy and toxicity
Epigenetics	DNA methylation and histone modification	↑ Potential improvement [35,43]	Regulation of immune-related gene expression	Target for combination therapy
(B)				
Microbiome	*Akkermansia muciniphila* enrichment	↑ Improved response [54,56]	Enhanced immune activation and T-cell priming	Modifiable biomarker
Microbiome	High microbial diversity	↑ Potentially improved response (context-dependent) [54,56]	Enhanced immune modulation	Potential predictive marker
Microbiome	Dysbiosis (e.g., antibiotic-induced)	↓ Reduced response [79,88]	Impaired microbial metabolite production and immune signaling	Avoidable risk factor
Metabolism	Obesity	Mixed effects (Obesity Pradox) [63,65]	Chronic inflammation and PD-1 upregulation	Context-dependent predictor
Metabolism	Hyperglycemia/diabetes	↓ Reduced response [66,68]	Immune dysfunction and systemic inflammation	Modifiable risk factor
Lifestyle	High-fiber diet	↑ Improved response [56,77]	Increased short-chain fatty acid (SCFA) production	Modifiable factor
Lifestyle	Physical activity	↑ Potential benefit [73]	Enhanced immune cell function and trafficking	Supportive intervention
Pharmacologic	Antibiotic use	↓ Reduced response [79,88]	Disruption of gut microbiota (dysbiosis)	Avoid near ICI initiation if possible
Pharmacologic	Baseline corticosteroid use	↓ Reduced response [91,93]	Systemic immunosuppression	Use cautiously
Pharmacologic	Proton pump inhibitor (PPI) use	↓ Potentially reduced response [81,89,90]	Altered gut microbiota composition	Monitor use

**Table 2:** Summary of Clinical Trials Evaluating Fecal Microbiota Transplantation (FMT) in Combination with Immune Checkpoint Inhibitors in Cancer.

Trial/Study	Cancer Type	Patient Population	Intervention	Key Outcomes	Key Findings
Phase I (early landmark, NCT not reported)	Melanoma	Anti-PD-1-refractory	FMT from responders + anti-PD-1	30% ORR (3/10), 1 CR	Microbiome modulation and increased tumor immune activation
NCT03341143	Melanoma	Anti-PD-1-refractory	FMT from responders	~20% ORR, ~40% disease control	Microbiota engraftment; increased CD8^+^ T-cell activity
NCT03772899	Advanced melanoma	Treatment-naïve	FMT from healthy donors + nivolumab/pembrolizumab	65% ORR, 20% CR	Safe; beneficial microbiome changes
NCT0495158	NSCLC & Melanoma	Advanced disease	FMT + anti-PD-1	80% ORR (NSCLC), 75% (melanoma)	Reduced harmful taxa; improved outcomes
NCT04264975	Advanced solid tumors	Anti-PD-1-refractory	FMT from responders	46.2% disease control	Increased cytotoxic T-cell activity
NCT05251389	Melanoma	Anti-PD-1-refractory	FMT from responders vs. non-responders	Ongoing	Evaluating differential clinical benefit
NCT04130763	GI cancers	PDL-1-resistant	FMT from healthy donors + anti-PD-1	20% ORR	Donor microbes linked to immune activation
MR3622010054	NSCLC	PD-1 secondary resistance	FMT + PD-1 rechallenge + chemotherapy	29.6% ORR	Increased microbial diversity and CD8^+^ T-cell infiltration

## Data Availability

No new data were created or analyzed in this study. Data sharing does not apply to this article.

## Abbreviations

The following abbreviations are used in this manuscript:

ORR	objective response rate
CR	complete response
FMT	fecal microbiota transplantation
NSCLC	non-small cell lung cancer
PD-1	programmed cell death protein 1
PD-(L)1	programmed death (ligand) 1
GI	gastrointestinal
ICI	immune checkpoint inhibitor
CD8^+^ T cells	cytotoxic T lymphocytes
NLR	neutrophil-to-lymphocyte ratio
HLA	human leukocyte antigen
SNP	single nucleotide polymorphism
MMR	mismatch repair
SCFA	short-chain fatty acid
PPI	proton pump inhibitor
